# Proteinaceous Pheromone Homologs Identified from the Cloacal Gland Transcriptome of a Male Axolotl, *Ambystoma mexicanum*

**DOI:** 10.1371/journal.pone.0146851

**Published:** 2016-02-17

**Authors:** Kevin W. Hall, Heather L. Eisthen, Barry L. Williams

**Affiliations:** Department of Integrative Biology and BEACON Center for the Study of Evolution in Action, Michigan State University, East Lansing, Michigan, United States of America; CNRS, FRANCE

## Abstract

Pheromones play an important role in modifying vertebrate behavior, especially during courtship and mating. Courtship behavior in urodele amphibians often includes female exposure to secretions from the cloacal gland, as well as other scent glands. The first vertebrate proteinaceous pheromone discovered, the decapeptide sodefrin, is a female attracting pheromone secreted by the cloacal gland of male *Cynops pyrrhogaster*. Other proteinaceous pheromones in salamanders have been shown to elicit responses from females towards conspecific males. The presence and levels of expression of proteinaceous pheromones have not been identified in the family Ambystomatidae, which includes several important research models. The objective of this research was therefore to identify putative proteinaceous pheromones from male axolotls, *Ambystoma mexicanum*, as well as their relative expression levels. The results indicate that axolotls possess two different forms of sodefrin precursor-like factor (alpha and beta), as well as a putative ortholog of plethodontid modulating factor. The beta form of sodefrin precursor-like factor was amongst the most highly expressed transcripts within the cloacal gland. The ortholog of plethodontid modulating factor was expressed at a level equivalent to the beta sodefrin precursor-like factor. The results are from a single male axolotl; therefore, we are unable to assess how representative our results may be. Nevertheless, the presence of these highly expressed proteinaceous pheromones suggests that male axolotls use multiple chemical cues to attract female conspecifics. Behavioral assays would indicate whether the putative protein pheromones elicit courtship activity from female axolotls.

## Introduction

Conspecific chemical cues have a well-documented and important role in vertebrate behavior, including mammals [1, 2], reptiles [3], fish [4], and amphibians [5]. Within salamanders, conspecific chemical cues are involved in the recognition of individuals [6], territoriality [7], and the modification of sexual receptivity [8]. The first peptide pheromone discovered was the decapeptide sodefrin, which is released by the cloacal gland of the male Japanese fire-belly newt, *Cynops pyrrhogaster* [9]. Since then, variants of sodefrin have been identified in other populations and species of *Cynops* [10, 11]. In newts that court in water, such as *Cynops* and *Lissotriton*, the male indirectly transfers the pheromone by wafting water currents towards the female’s nares via tail fanning movements [12]. Within the family Plethodontidae, which exhibit terrestrial courtship displays, at least three peptide pheromones are secreted from the mental glands located under the chins of males. Direct transfer of pheromones during courtship occurs either when a male’s mental gland makes contact with the female’s nares, or when the pheromones are scratched into the female’s skin with the male’s premaxillary teeth [8, 13, 14, 15].

Most protein pheromones related to courtship behavior in salamanders are comprised of at least one protein domain belonging to the three-finger domains (TFD) superfamily, which also contains uPar, Ly-6, CD59, and a number of snake toxins [16]. TFDs are so named because they contain at least one tertiary domain structure of three loops extending from a hydrophobic core that is cross-linked by disulfide bridges [17]. TFD-containing proteins are common across vertebrates and often include a signal peptide for their exportation via the Golgi apparatus and transport vesicles. In addition, the number of TFDs per protein can vary, and the protein may either be membrane bound via a glycophosphatidylinositol (GPI)-anchor or excreted without a GPI-anchor in soluble proteins [17]. The TFD has a strongly conserved cysteine motif with a high rate of sequence evolution in the intervening loop regions [16]. The function of any one TFD is often tissue specific. For example, the long- and short-chain neurotoxins and cardiotoxins found in snake venom are comprised of one TFD and lead to membrane disruption [16]. However, the complement regulatory protein CD59 has a similar structure to snake toxins, but has a GPI-anchor and functions as an inhibitor of complement-mediated lysis [18]. Preprosodefrin (PPS), sodefrin’s precursor protein, is comprised of a signal peptide, a TFD and a low complexity 63-amino acid carboxyl-terminal region. This low complexity region lacks any cysteine disulfide bridges and is cleaved by prohormone convertases to produce the biologically active conspecific female attractant, sodefrin [19]. Expression of PPS and biological activation of sodefrin in *C*. *pyrrhogaster* is regulated by androgen and prolactin [20].

Because PPS lacks a GPI-anchor, the gene falls within the group of TFD-containing proteins that include the aforementioned snake toxins. Comparative genomic analyses indicate that, within salamanders, PPS is a derived homolog of an ancient salamander pheromone, sodefrin precursor-like factor (SPF) [21]. SPF contains an 18-amino acid signal peptide, a complete TFD, a partial TFD, and a short low complexity carboxyl-terminal region [14]. Janssenswillen *et al*. [22] used molecular evolutionary approaches to split SPF-like genes into two groups, alpha SPF and beta SPF. Alpha SPF contains an amino-terminal eight-cysteine TFD ending with a XCXXXXCN motif, followed by a six-cysteine partial TFD. Beta SPF contains an animo-terminal ten-cysteine TFD ending with a CCXXXXCN motif, followed by an eight-cysteine partial TFD. Furthermore, beta SPF homologs within *Cynops* exhibit an additional 62-base pair (bp) insert between the first complete TFD and the second partial TFD, which comprises PPS. This insert resulted in the loss of the partial TFD by frameshift mutation and the creation of the low complexity tail on the carboxyl end, from which sodefrin is cleaved. An additional proteinaceous pheromone, plethodontid modulating factor (PMF), was discovered in the mental gland of *Plethodon shermani* and is comprised of only a twenty-amino acid signal peptide, a complete TFD with a carboxyl CCXXXXCN motif [15]. Again, the cysteine motif is highly conserved, yet the intervening composition of amino acids evolved rapidly. The evolutionary relationships among the peptide pheromones SPF, PPS, and PMF, is not clear [22]. Finally, plethodontid receptivity factor (PRF) is an additional peptide pheromone that appears to be unique to plethodontid salamanders. PRF lacks the cysteine spacing pattern commonly found in the TFDs of SPF, PMF, or PPS and thus is not part of the same TFD superfamily, but instead is a member of the interleukin-6 cytokine family [23].

The axolotl, *Ambystoma mexicanum*, is a great model salamander to further develop an understanding of the neural mechanisms underlying chemical communication in amphibians [24, 25]. Axolotls are neotenic and fully aquatic, naturally distributed in lakes in central Mexico [26, 27]. Courtship behavior in male axolotls involves nudging the female with the snout and making undulating motions with the tail. The male and female place their snouts near each other’s cloacae, and as the male moves away from the female, the female follows the male with her head near his cloaca. The female sometimes nudges the male near the cloaca with her snout. The male deposits a spermatophore that consists of a packet of sperm atop a gelatinous base and leads the female over the spermatophore. As the male moves away from the spermatophore, the female follows the male until her cloaca comes into contact with the spermatophore and then she picks up the sperm packet with her cloaca [28, 29]; fertilization occurs internally [30]. This courtship pattern is broadly similar across many salamanders [28]. In addition, conspecific chemical cues produced by glands have been shown to elicit courtship behavior in several salamander species [8, 9, 12, 20].

Given the apparent importance of the male cloaca during courtship and the potential for expression of multiple peptide pheromone homologs, we assembled a transcriptome from the male cloacal gland. Transcriptomic data will facilitate identification and measurement of relative expression levels among alpha SPF, beta SPF, PPS, PMF, and PRF orthologs, providing a foundation for prioritizing future studies of pheromones in this species. Specifically, our goals were to address the following three questions: Which orthologs are expressed in the male cloacal gland? How many members of each gene family are expressed in the male cloacal gland? Are there clear differences in the levels of expression amongst orthologs?

In this study, the cloacal gland transcriptome from a single male axolotl was assembled to identify which orthologs are expressed at a single point in time. The putative contigs from the transcriptome were filtered, mapped, and functionally annotated to determine the number of contigs within each gene family (PPS, PMF, SPF, and PRF) expressed within the cloacal gland. Expression levels for the filtered contigs were calculated and any clear differences in the levels among the orthologs associated with amphibian courtship behavior were identified. The results indicate that male axolotls possess two different forms of SPF (alpha and beta [22]) as well as a putative ortholog of PMF. The beta form of SPF was amongst the most highly expressed transcripts within the cloacal gland and the ortholog of PMF was expressed at a level equivalent to the beta SPF. These results are from the sampling of one individual and therefore the expression estimates may not accurately represent male axolotl expression levels as a whole. Nevertheless, the presence of these highly expressed proteinaceous pheromones may indicate that male axolotls use several chemical cues to attract female conspecifics. We present here a fully annotated transcriptome, providing an important resource for the axolotl research community.

## Materials and Methods

### Animal maintenance and tissue collection

The subject was obtained from the federally-funded Ambystoma Genetic Stock Center (University of Kentucky, Lexington, KY) that breeds axolotls and other salamanders for the research community. In our lab, the axolotl was maintained in recirculated, filtered Holtfreter’s solution (60 mM NaCl, 2.4 mM NaHCO_3_, 0.67 mM KCl, 0.81 mM MgSO_4_, and 0.68 mM CaCl_2_ in dH_2_O (pH 7.5)) at 20°C. The photoperiod of the room was altered each month to match that of the animal’s native habitat in Mexico City, MX. The axolotl was fed commercial salmon chow (Rangen, Buhl, Idaho, USA) three times each week. All experimental conditions were approved by and carried out in accordance with the Michigan State University’s Institutional Animal Care and Use Committee recommendations (approval no. 10/12-195-00).

A wild-type adult male axolotl in breeding condition was sacrificed by rapid decapitation followed by pithing and the cloacal gland was dissected out at the morphological boundaries that define glandular tissue from the surrounding non-glandular tissue. The entire gland was immediately frozen in liquid nitrogen, ground into a powder with a mortar and pestle, and stored at -80°C until RNA extraction.

### RNA-Seq preparation and sequencing

Total RNA was extracted from the axolotl cloacal gland tissue using RNeasy Mini Kit reagents (Qiagen, USA) according to manufacturer instructions. Residual DNA was removed using DNase I digestion and cDNA was constructed using the Promega cDNA kit. Briefly, total RNA (1 μg) was reverse-transcribed at 42°C into DNA using 5 U of reverse transcriptase, 1x RT buffer, 1 mM of deoxyribonucleotide triphosphate, 10 ng/μl of random primer, and 20 U RNase inhibitor in a 20 μL reaction. RNA quality and quantity were assessed with an Agilent 2100 BioAnalyzer. Approximately 20 μg of cDNA was used for sequence analysis via Illumina HiSeq™ 2000 platform at the Michigan State University Research Technology Support Facility (East Lansing, MI) to generate 50 bp single-end reads. The raw data from Illumina HiSeq were deposited in the NCBI Short Read Archive (SRA) database (Accession: SRR2140729).

### RNA-Seq data trimming, read filtering and digital normalization

Trimmomatic v.0.32 [31] was used to remove the single end adapters from the Illumina (FASTQ) data. Low quality reads or bases were removed using FastQ Quality Control Software v.1.3 (FaQCs) [32]. FaQCs was used to perform a low-quality filtering process where reads with a phred quality score lower than 20 were discarded. Bases with a phred quality score less than 20 were trimmed from the 5’ and 3’ ends of the reads. Reads with lengths that dropped below 35 bp were discarded. All retained reads comprised the ‘filtered reads’ database.

The filtered reads were digitally normalized to facilitate rapid transcriptome assembly. The program khmer v.1.3 and the short read sequence utility screed v.0.7 [33] executed the python script ‘normalize-by-median.py’, which reduced the number of redundant reads to twenty reads per k-mer. The k-mer length used to determine the maximum number of reads with repetitive sequences was twenty, based on recommendations from Brown *et al*. [34].

### RNA-Seq transcriptome assembly and contig filtering

Transcriptome assembly was carried out using the program Trinity v. r20140413p1 [35]. The assembly parameters were set to—min_contig_length 200,—JM 48G,—glue_factor 0.01, and—min_iso_ratio 0.1, based upon the recommendations from Li *et al*. [36]. The program TransDecoder v.2.0.1 [37] translated predicted protein sequences from the assembled transcriptome by identifying open reading frames (ORFs) coding for at least 125 amino acids.

Putative proteins within contigs were used as queries to search three protein databases using BLAST+ v.2.2.30 [38]: *Xenopus tropicalis* Protein (ftp://ftp.xenbase.org/pub/Genomics/Sequences/xtropProtein.fasta), UniProtKB Swiss-Prot (ftp://ftp.uniprot.org/pub/databases/uniprot/current_release/knowledgebase/complete/uniprot_sprot.fasta.gz), and Uniref–Uniref90 (ftp://ftp.uniprot.org/pub/databases/uniprot/uniref/uniref90/uniref90.fasta.gz). All three databases were downloaded on March 20, 2015. Contigs that had at least one match with an e-value of at least 10^-5^ from the BLASTx or BLASTp searches were retained for further analyses and comprised the ‘filtered transcriptome assembly’. Bowtie2 v.2.2.3 was used to align the filtered reads to the filtered transcriptome assembly as a measure of assembly quality [39]. To facilitate further discovery of potential contigs, the reads that Bowtie2 could not align to the filtered transcriptome were reassembled and putative ORFs were identified as previously described. The filtered contigs from this unaligned read assembly were added to the existing data and duplicate contigs were removed, resulting in the final assembly. This Transcriptome Shotgun Assembly project has been deposited at DDBJ/EMBL/GenBank under the accession GEBK00000000. The version described in this paper is the first version, GEBK01000000.

### Transcript expression and functional annotation

Functional annotation was carried out using Interproscan v.5.11–51.0, BLASTx or BLASTp to generate xml formatted files of all matches and their corresponding contigs from the final assembly, which were subsequently analyzed using BLAST2GO v.3.0.10 [40]. The top matches from BLASTx or BLASTp were retained from the *Xenopus* genome database. If there was no match to the *Xenopus* database then the best match from the Refseq database was retained. This matching process was continued using databases in the following order: UniProtKB Swiss-Prot, Uniref90, and NR protein databases. BLAST2GO was then used to map and annotate the sequences with the associated GO terms describing biological processes, cellular components, and molecular functions. The sequences with corresponding Enzyme Commission (EC) numbers obtained from BLAST2GO were mapped to the Kyoto Encyclopedia of Genes and Genomes (KEGG) to determine metabolic pathways that correspond with putative gene functions [41]. RSEM-eval from the DETONATE software package [36] was used to estimate the abundance of transcripts by mapping the filtered reads back to the final assembly. Expression levels were calculated as Transcripts Per Million (TPM) with 95% confidence intervals. Descriptions of all custom scripts used for these analyses are included in the S1 Code.

## Results

### Illumina HiSeq reads, read filtering, read assembly, assembly filtering

**Fig 1:**
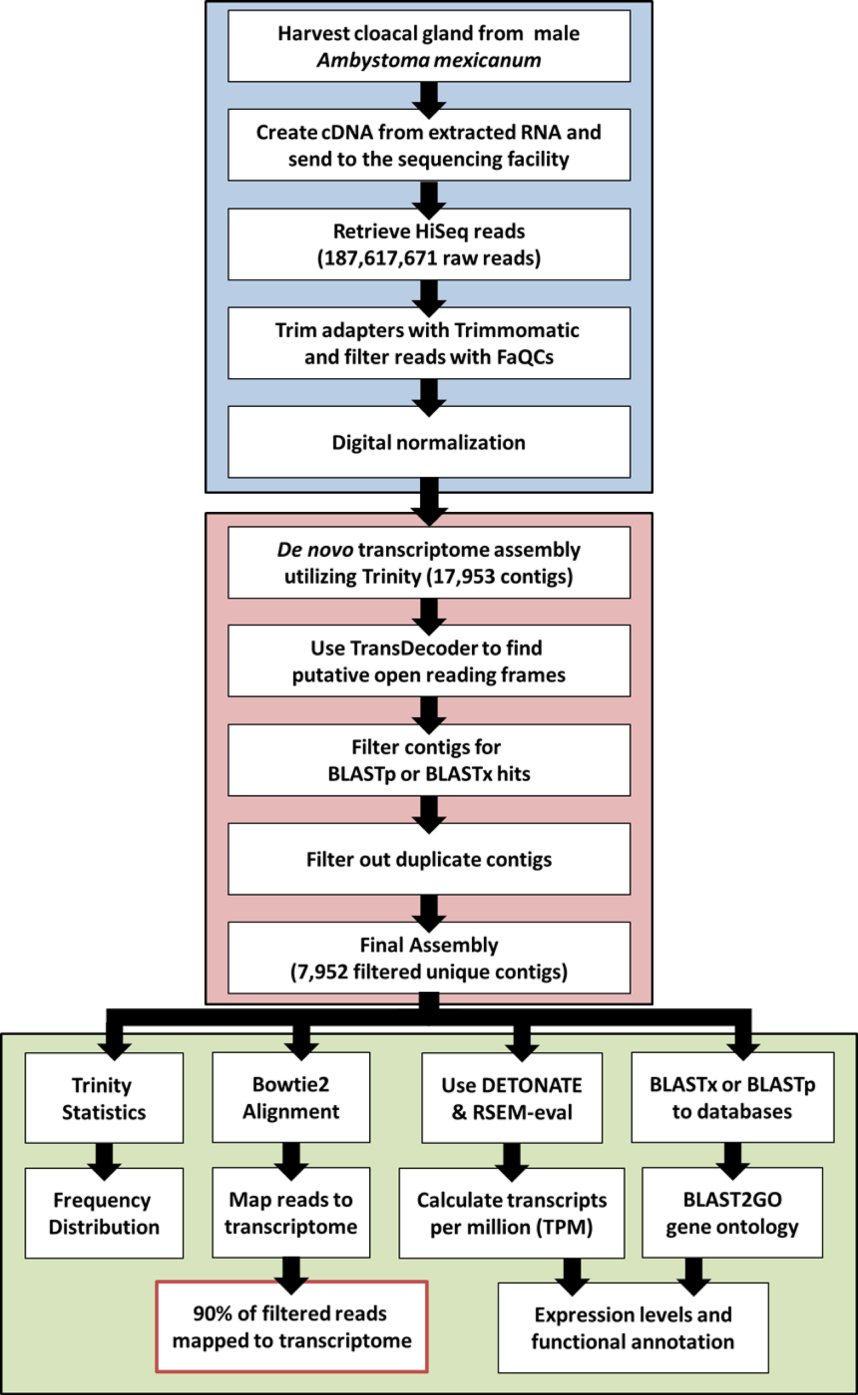
Pictorial representation of the process to assemble a transcriptome. First, RNA was extracted from the cloacal gland and HiSeq Illumina reads were created. The sequence reads were parsed and filtered for quality and removal of adaptor sequences (blue). Next, *de novo* assembly was generated and the transcripts were filtered based upon BLAST hits and redundant contigs were removed (red). Reads were mapped back to contigs, genes were annotated, and gene ontology was applied using BLAST and BLAST2GO (green). Finally, an analysis of the assembly and the quantity and distribution of transcripts was performed.

The workflow for bioinformatics analyses is illustrated in Fig 1. After removal of the sequence adapters, the Illumina single-end sequencing produced 187,617,671 reads of 50 bp each, totaling 9.38 gigabases (Gb) (Table 1). FaQCs filtered out 3,824,501 (2.04%) reads with a quality score less than 20, resulting in 183,793,170 reads that were trimmed and filtered (97.96% of the total raw reads). The final filtered and trimmed FastQ file contained 89.27% of the total reads with a quality score greater than or equal to 30. The GC content of the filtered data was 53.98%. The mean read length (± standard deviation) after filtering was 49.42 ± 2.02 bp. Redundant reads were removed through digital normalization resulting in 19,074,717 retained reads with a mean length of 49.56 ± 1.67 bp. The Trinity assembly of all of the filtered reads resulted in 17,953 contigs and a total length of 9,653,916 bp with a GC content of 45.56%. The N50 was 696 bp with a mean length of 537.73 ± 930.9 bp. The final assembly resulted in 7,952 contigs and a total length of 5,377,213 bp with a GC content of 47.48%. The N50 was 1,143 bp with a mean length of 676.21 ± 1246.3 bp.

**Table 1:** Summary of sequencing data and assembly of the male axolotl cloacal gland transcriptome.

Category	Raw Data	Filtered Raw Data	Digital Normalization	Assembly	Final Assembly
Total Reads	1.87 x 10^8^	1.83 x 10^8^ (98% of raw reads)	1.9 x 10^7^ (10% of raw reads)		
Total Bases (Gb)*	9.4	9.1	0.9		
Mean read length (bp^§^ ± S.D. ^¥^)	50.0 ± 0.0	49.4 ± 2.0	49.6 ± 1.7		
GC content	54%	53.98%	46.97%	45.56%	47.48%
Contigs > 200 bp				17,953	7,952
N50				696 bp	1,143 bp
Average length ± S.D.				537.7 ± 930.9 bp	676.2 ± 1246.3 bp
Total length				9,653,916 bp	5,377,213 bp

* gigabases (Gb)

^§^ base pairs (bp)

^¥^ standard deviation (S.D.)

### Assembly analysis

**Fig 2:**
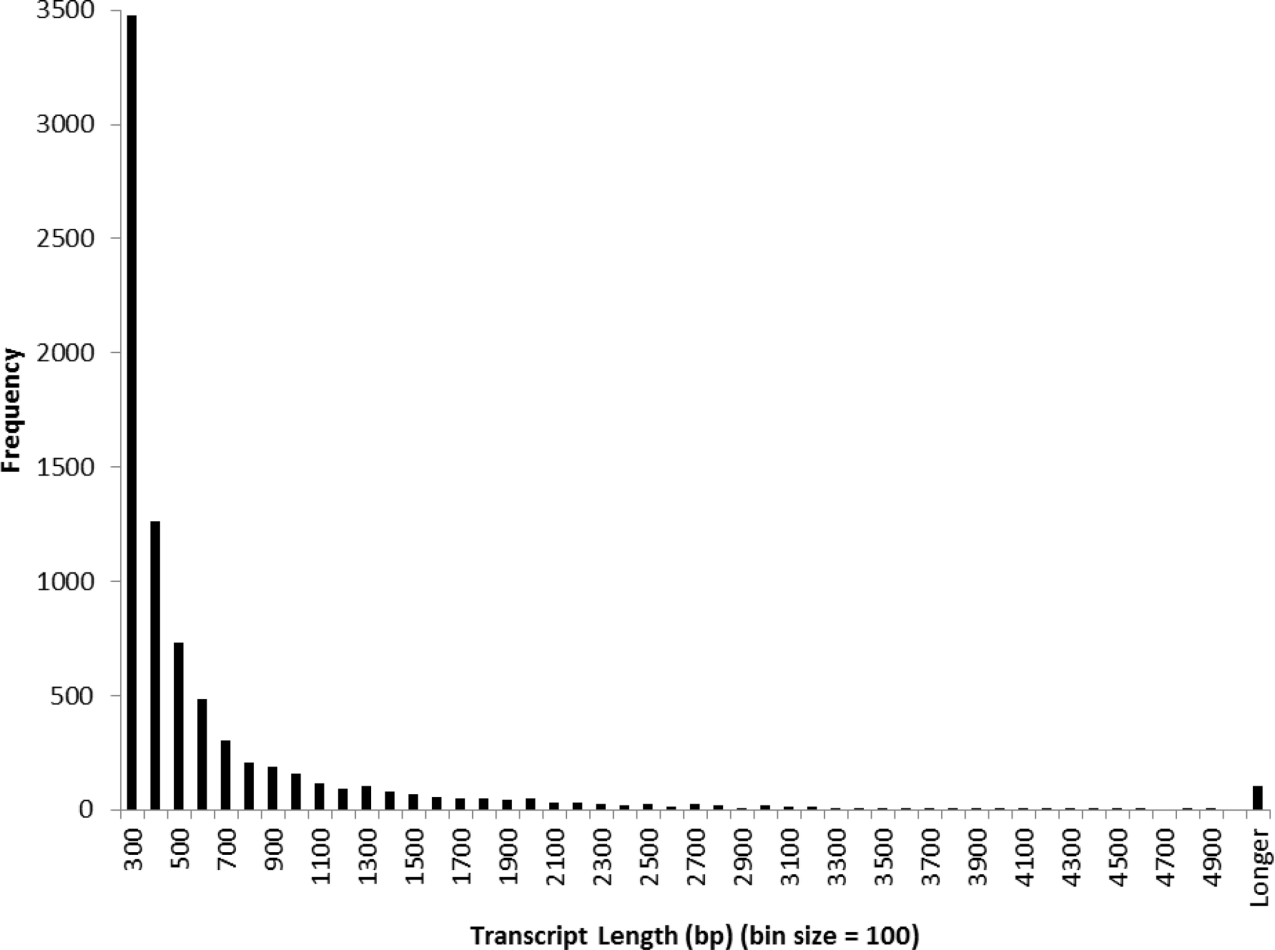
Distribution of sequence lengths from the final transcriptome assembly. The x-axis indicates sequence sizes from 200 bp to ≥ 5000 bp with a bin size of 100; the y-axis indicates the number of transcripts for a given sequence length bin. Most (60%) of the final assembly sequences range between 200 399 nucleotides in length; however, more than 6% of transcripts are longer than 2000 bp.

The distribution of sequence lengths from the contigs is displayed in Fig 2. Of the 7,952 contigs obtained, 5,465 (68.7%), 2,000 (25.2%) and 487 (6.1%) were distributed between 200–599 bp, 600–1999 bp, and greater than or equal to 2000 bp, respectively. The longest transcript was 36,996 bp and sixteen transcripts were longer than 11,000 bp. Quality was measured by the percent of reads that Bowtie2 aligned to the final assembly. Bowtie2 aligned 90.2% of the filtered raw reads to the final assembly. TransDecoder identified putative proteins of at least 125 amino acids from the filtered transcripts, resulting in a predicted 3,168 (39.8%) contigs with ORFs meeting the minimum length criteria.

**Fig 3:**
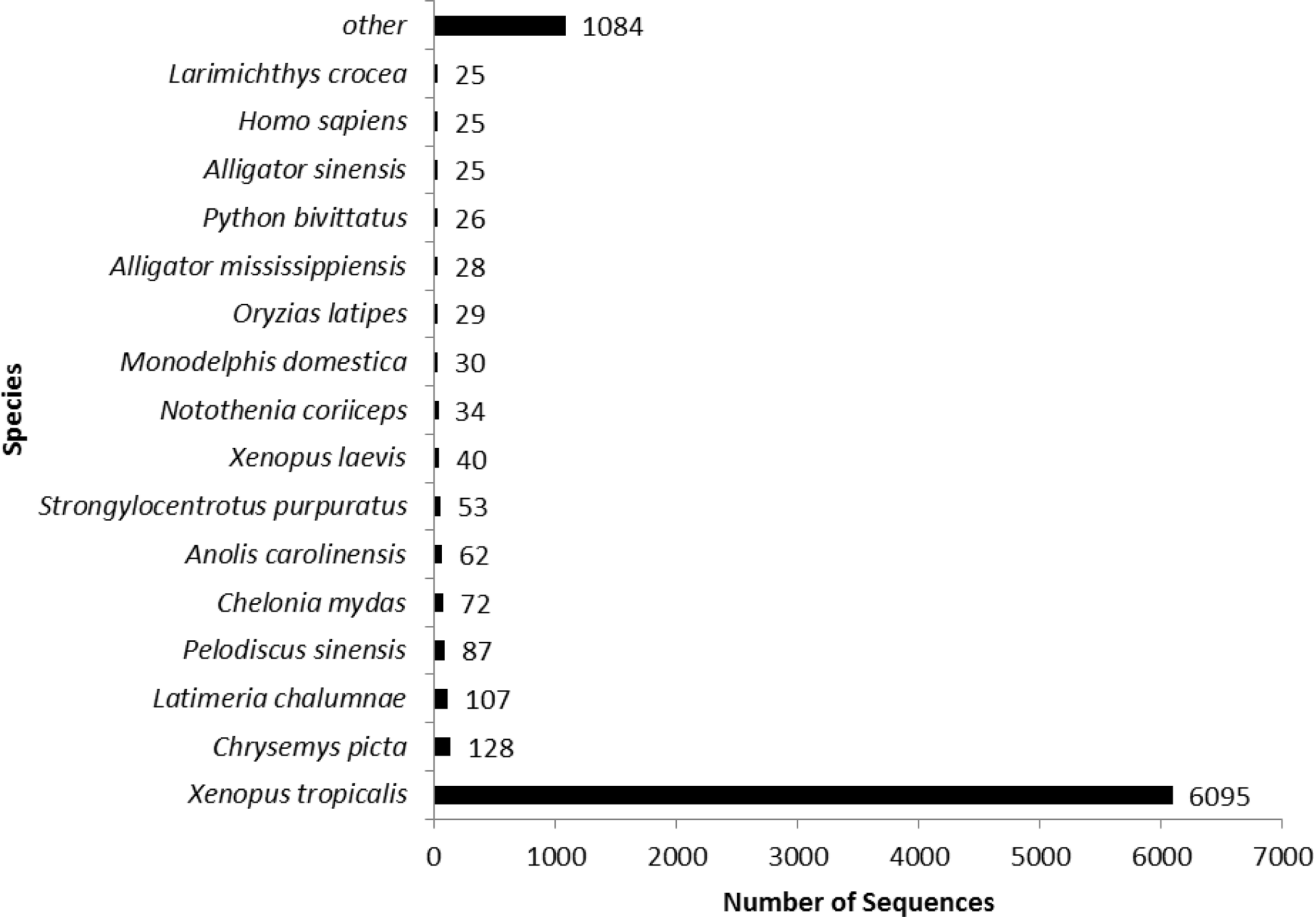
BLASTx or BLASTp top-hit species distribution generated from matches to the *X*. *tropicalis*, UniProtKB, and UniRef90 protein databases.

The top-hit species distribution generated from BLASTx and BLASTp against the *X*. *tropicalis*, RefSeq, UniprotKB, UniRef90, and NR protein databases is displayed in Fig 3. As a result of the filtering process, all of the 7,952 transcripts had at least one hit against the *X*. *tropicalis*, UniprotKB or Uniref90 protein databases, which corresponds to 38% of the approximately 21,000 known protein-coding sequences in the *X*. *tropicalis* genome [42]. Of the 7,952 contigs, 6,095 had a best match within the *X*. *tropicalis* database; however, forty genes received their only match (or statistically stronger match) within the *X*. *laevis* database.

**Fig 4:**
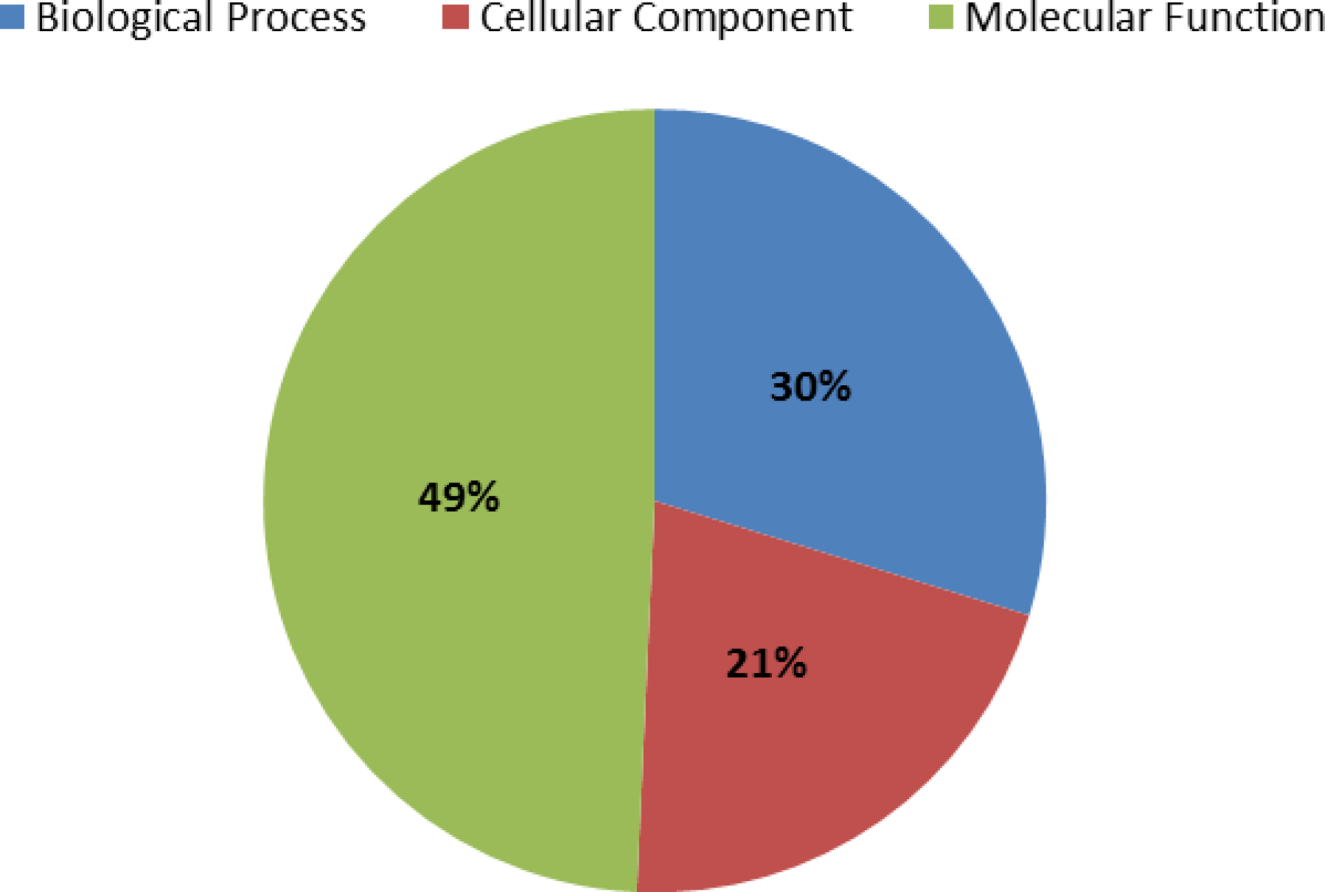
Distribution of GO annotations among biological processes, cellular components and molecular function.

**Fig 5:**
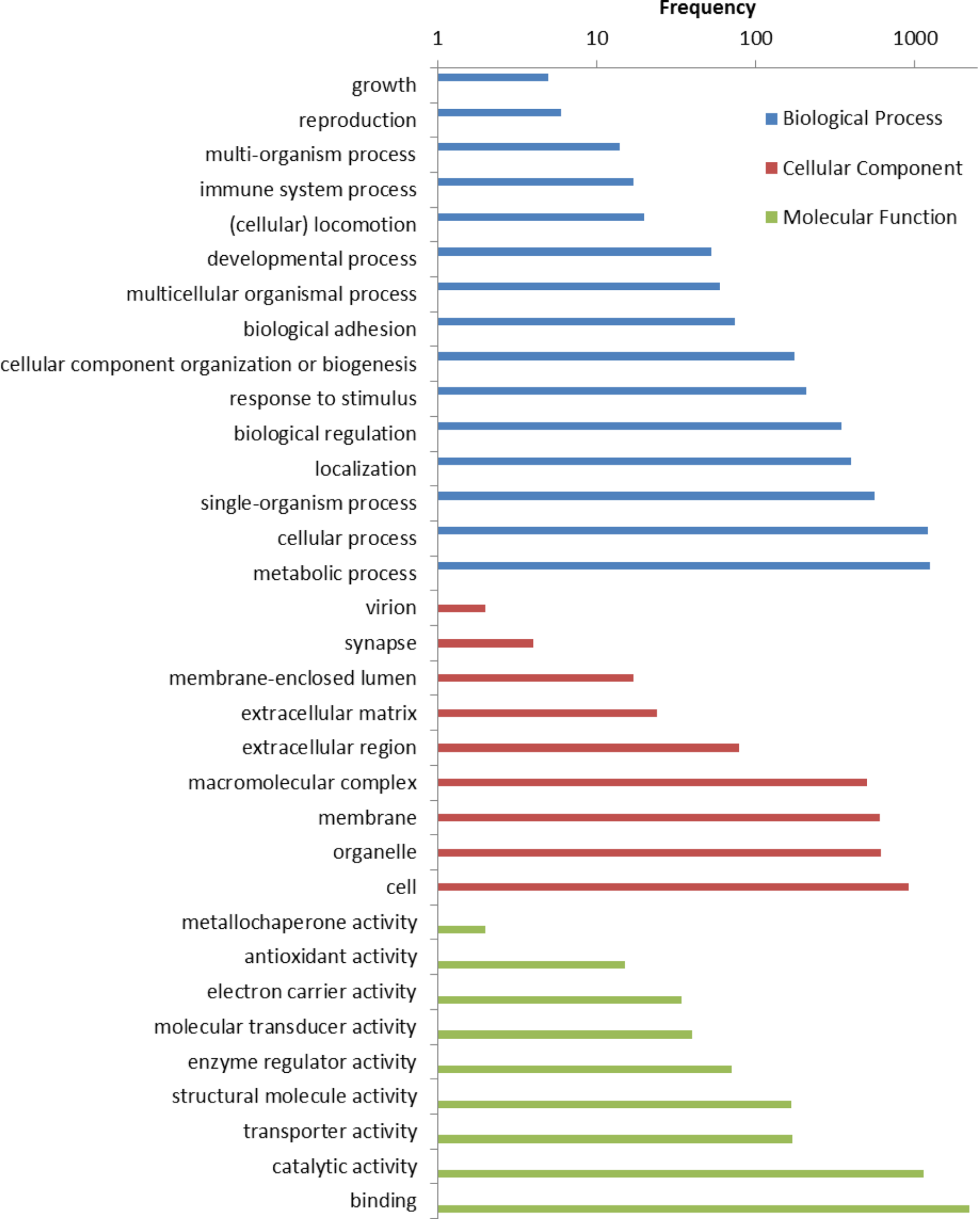
Level two GO classifications of final assembly contigs. The results are summarized in three main categories: biological process (blue), cellular component (red), and molecular function (green). The x-axis indicates the number of genes in a category on a log scale. The y-axis on the left indicates the specific category of genes in that main category.

A total of 5,261 (66.2%) contigs received at least one gene ontology (GO) term. A total of 17,706 GO terms, across fifteen GO levels, was applied to the sequences and classified into one of three level-one GO terms: biological process, cellular component, or molecular function (Fig 4). Biological processes represented 49%, cellular components represented 21%, and molecular functions represented 30% of the GO terms. Within the biological processes, “metabolic process” and “cellular process” represented the most abundant level-two GO annotations, occurring 1,242 and 1,219 times, respectively. Among the cellular component category, “cell” was the most abundant GO annotation, while “binding” and “catalytic activity” were the most abundant GO annotations in the molecular function category. The level-two GO classifications of the contigs are presented in Fig 5. Finally, 1,504 contigs were grouped into 99 KEGG pathways. The most highly represented pathways were ‘purine metabolism’ (302 contigs, 20.1%), ‘thiamine metabolism’ (244 contigs, 16.2%), and ‘biosynthesis of antibiotics’ (108 contigs, 7.2%).

**Fig 6:**
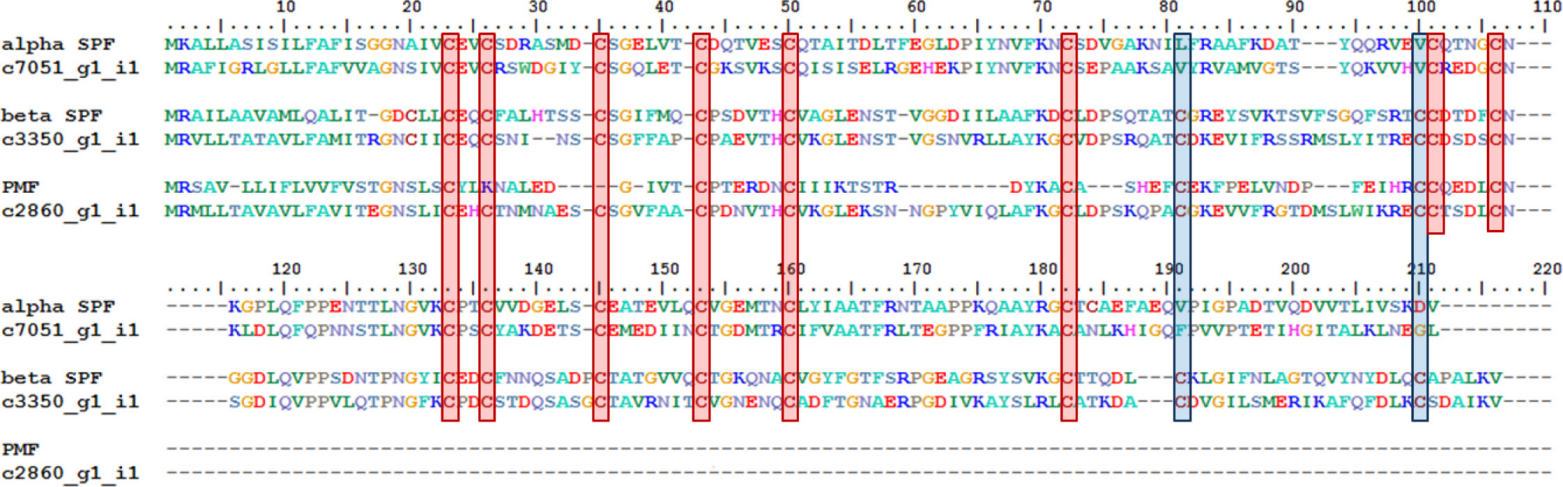
Comparison of deduced amino acid sequences for alpha SPF, beta SPF, and PMF aligned using ClustalW Multiple Alignment. Alpha SPF sequence from *Notophthalmus viridescens* aligned with the most highly expressed homolog, contig c7051_g1_i1 which was expressed at 35 TPM. Beta SPF sequence from *Lissotriton vulgaris* aligned with the most highly expressed homolog, contig c3350_g1_i1 which was expressed at 1477 TPM. PMF sequence from *Plethodon shermani* aligned with the most highly expressed homolog, contig c2860_g1_i1 which was expressed at 1336 TPM.

**Fig 7:**
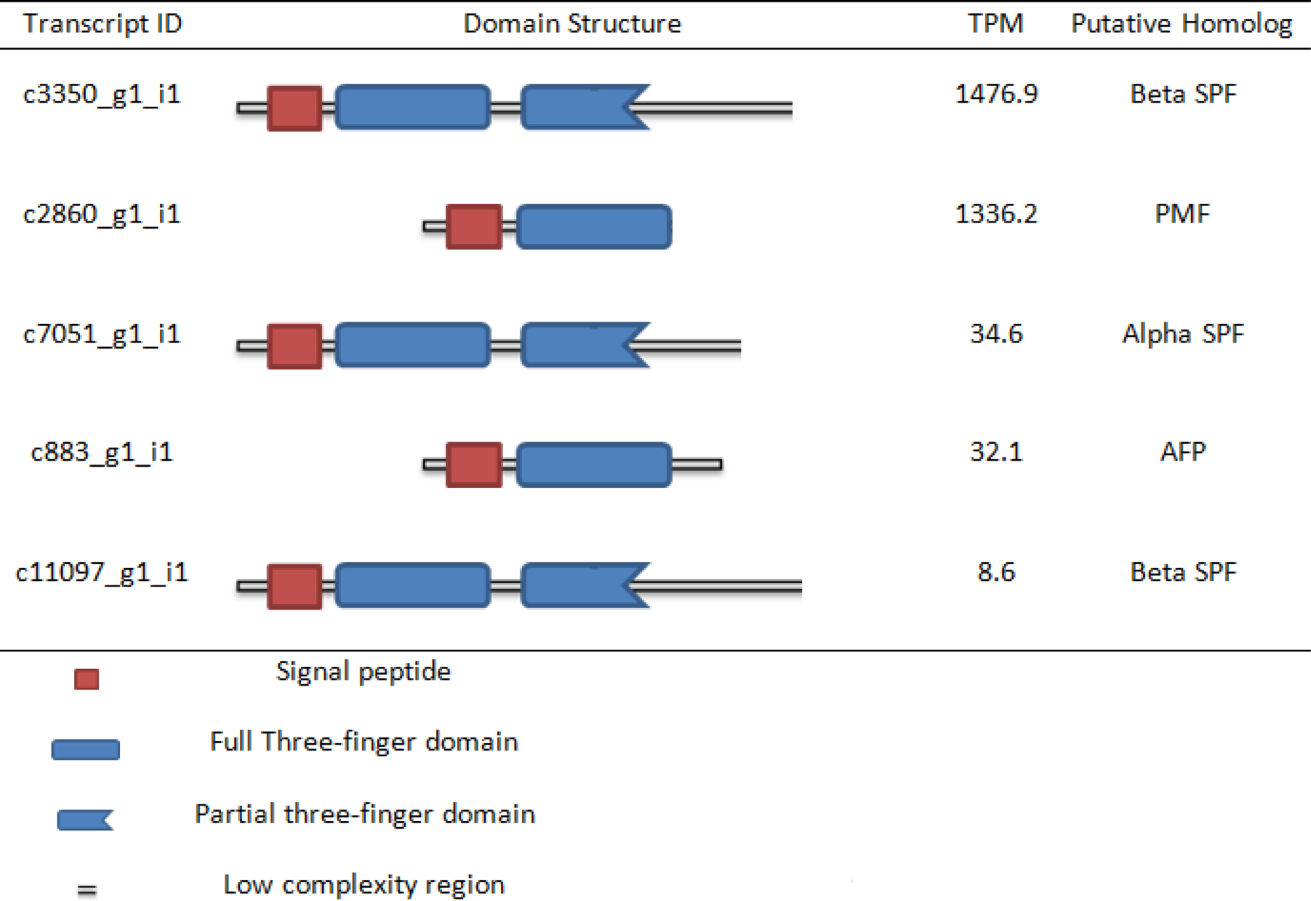
Putative full length homologs of alpha SPF, beta SPF, PMF, and AFP.

The homologs of PPS, SPF, and PMF were identified by their characteristic domain structure, as well as the characteristic pattern of spacing among cysteines within their three finger domain(s) (Fig 6). These patterns, as well as significant BLAST scores and Interproscan matches, were used to identify all putative protein pheromone homologs in the final assembly (Fig 7). An alignment of the putative homolog to SPF is shown in Fig 6. A PPS homolog was not found when using strict criteria, which included a signal peptide, a complete TFD, and a low complexity region at the carboxyl-terminus. Ten SPF-like homologs were identified that contained a signal peptide at the amino-terminus, followed by one full TFD, a partial TFD, and a short low complexity region. Three PMF-like homologs were characterized by a signal peptide, one TFD, and a very short or nonexistent low complexity region. Five homologs to a previously discovered axolotl antifreeze protein (AFP) also were identified, but the sequence exhibited a pattern also consistent with PMF-like homologs [43]. Gene IDs, domain structure, TPM, and putative homologs are summarized in Fig 7.

**Fig 8:**
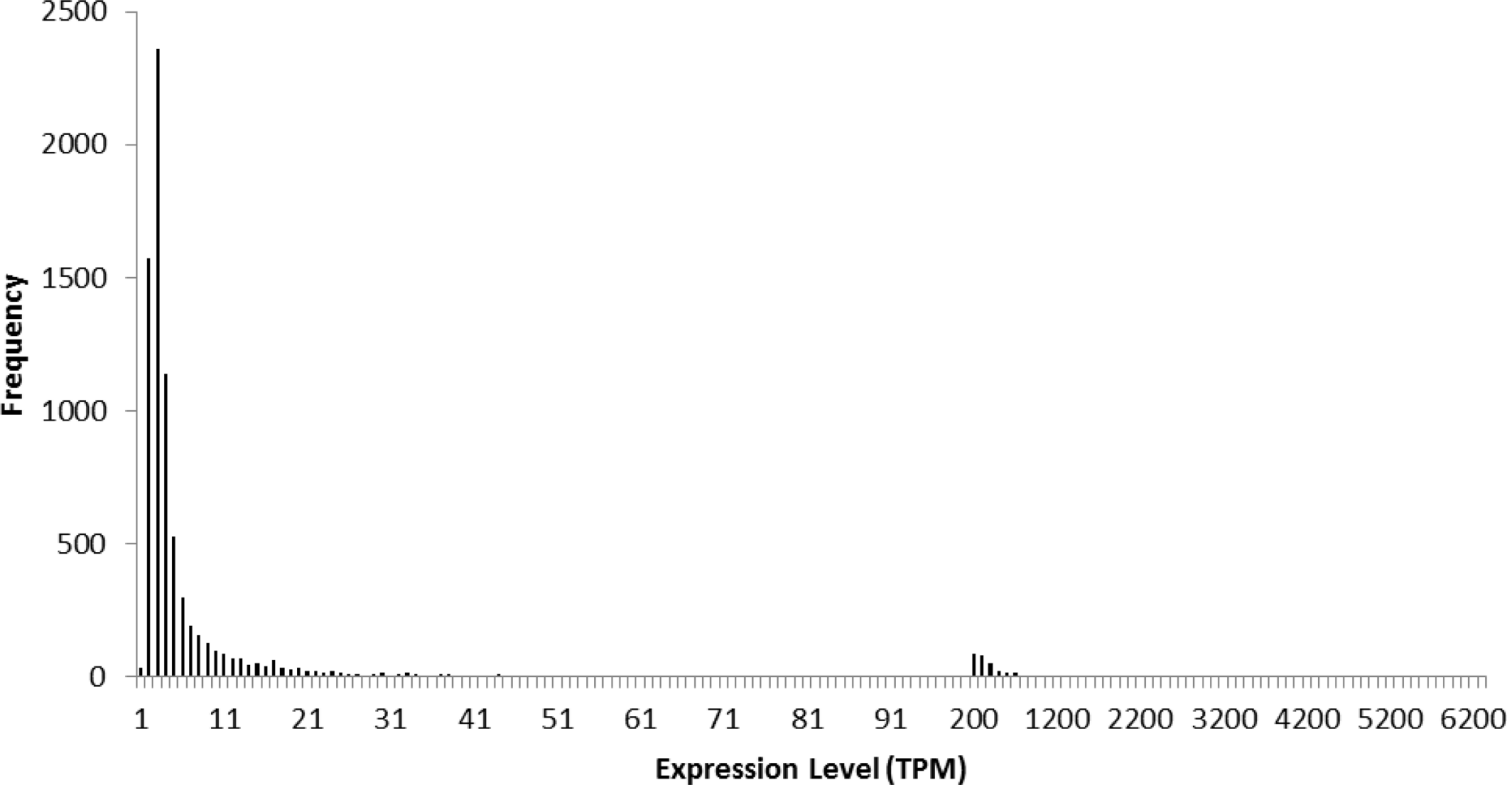
Frequency distribution of expression levels of putative genes expressed in transcripts per million (TPM). The x-axis indicates expression levels from 0 TPM to 100 TPM with a bin size of 1 and from 101 to ≥ 6200 with a bin size of 100; the y-axis indicates the number of transcripts for a given TPM bin.

The number of contigs with at least two TPM, the minimal TPM considered biologically active, was 6,637 (Fig 8). Nineteen contigs exhibited an expression level greater than 1,000 TPM. Beta SPF was the 17^th^ most highly expressed transcript at 1476.9 ± 6.4 (mean ± 95% confidence interval) TPM. A putative homolog of PMF was the 18^th^ most highly expressed transcript at 1336.2 ± 15.0 TPM. A number of other cysteine-rich secretory proteins were highly expressed, but the spacing among cysteines was not consistent with a canonical three-finger domain pattern. Additionally, ribosomal proteins are typically highly expressed, but in this tissue only one was expressed at a level greater than beta SPF.

## Discussion

### Ilumina single-end sequencing and assembly

With the development of next generation sequencing, transcriptome data from non-model organisms have grown exponentially. Nevertheless, most non-model organisms lack a well annotated reference genome. Quality axolotl transcriptome assemblies exist. Recent studies producing axolotl expressed sequence tags derived from limb regeneration and brain samples resulted in transcriptomes containing 11,927 and 15,384 contigs [44, 45]. An assembly of approximately 17,000 contigs is located on Sal-Site (www.ambystoma.org) [46]. However, to date none of the available transcriptomes include the cloacal gland, which was the target of our investigation. At present, there is no single standardized method to assess the quality of a *de novo* transcriptome assembly; thus, different metrics are used in different studies. In this study, approximately 187 million single-end reads (9.38 Gb) were generated and assembled into 17,953 contigs. From this assembly, a total of 7,952 contigs (44.3%) had a BLASTx or BLASTp match, with a mean contig length of 676.2 ± 1246.3 bp. These contigs comprised the final assembly and their mean length is comparable to that of previous studies using Trinity as a *de novo* transcriptome assembler [47, 48]. The final assembly had an N50 score of 1,143 bp and a total of 1,147 contigs had lengths greater than 1,000 bp (14.4% of the filtered assembly), comparable to other salamander transcriptome assemblies [47]. According to Ramskold *et al*. [49], approximately 60–70% of genes in the genome are expected to be expressed in any particular cell type. If axolotls possess approximately 21,000 genes, similar to the well annotated *X*. *tropicalis* genome, then 38% of axolotl genes were detected. Thus, more than half of the genes predicted to be expressed in the male axolotl cloacal gland were identified in this study. Alternatively, this tissue may express an unusually small number of genes when compared to other axolotl transcriptome studies [44, 45], indicative of a highly specialized tissue. Further independent transcriptomic analyses will be required to distinguish between these competing hypotheses.

The contigs without BLAST matches may have been derived from untranslated regions, non-coding RNAs, sequences not containing a known protein domain, or may represent genes unique to axolotls. These unmatched contigs could provide a resource for identifying novel genes expressed in the cloacal gland of male axolotls. Of particular interest is the large number of reads that mapped back onto the final assembly. Read alignment is an important determinant of assembly quality; the higher the percentage of read alignment after transcript filtering, the better the assembly. Bowtie2 mapped greater than 90% of the filtered raw reads onto the final assembly, indicating that most reads were used to assemble the contigs with BLAST hits. This high percentage, in conjunction with the percentage of matched contigs, contig length, and N50 score, provides a high level of confidence that the assembly is accurate and consistent with comparable *de novo* transcriptome assemblies.

### Functional annotation of contigs

Because axolotls lack a well annotated reference genome, it is difficult to predict the potential functions encoded by the cloacal gland transcripts; therefore, various protein databases were used to help identify putative functions based on homology. The results indicate that 6,095 contigs (76.6%) have a putative homolog in the *Xenopus* protein database. The remaining 1,857 contigs (23.4%) have a putative homolog in the RefSeq, UniProtKB Swiss-Prot, Uniref90, or NR protein databases. A large proportion of contigs were assigned a GO term: 5,261 contigs (66.2%) had at least one GO term distributed among a wide array of GO categories [47]. The breakdown of many GO categories is comparable to other transcriptome assemblies [47, 48], except for the relatively high number of contigs (108 contigs (7.2%)) annotated within the “biosynthesis of antibiotics” category. These transcripts may be expressed as a mechanism to control bacteria living within the cloaca or on the skin of axolotls, assisting in maintaining the microbiome of the host organism or preventing infection from invasive microorganisms [50].

### Pheromone homologs in the male axolotl cloacal gland

The cloacal gland plays an important role during courtship in axolotls, similar to that of *C*. *pyrrhogaster* or to the mental gland of *P*. *shermani* [8, 11, 15, 19]. Given that proteinaceous pheromones are present in organs related to courtship in closely related species [22, 51], it is not surprising to find putative homologs identified based upon their primary sequence and domain structures. The orthology amongst PPS, SPF, and PMF is unclear; however Janssenswillen *et al*. [22] showed that PPS and SPF are paralogs following a gene duplication of SPF into alpha SPF and beta SPF. In this study, putative homologs to both alpha and beta SPF, as well as PMF, were identified in axolotls; however, no putative homologs to PPS were identified. In addition, putative androgen and prolactin receptors in the cloacal gland were identified, which were shown to regulate synthesis of proteinaceous pheromones in *Cynops* [20]. Although alpha SPF has been previously identified in axolotls [22], this is the first time a beta SPF has been identified in the species. Additionally, this is the first time a homolog to PMF has been identified outside of the family Plethodontidae. One homolog to PRF was identified by Interproscan, based upon the PRF domain, but the contig was much shorter than and poorly aligned to PRF and thus was considered non-orthologous to PRF.

The SPF-like contigs contained a signal peptide at the amino-terminus, followed by a complete TFD and a partial TFD. The partial TFD of the SPF-like contigs contained eight of the twelve cysteines found within the anterior TFD and notably lacked the final CCXXXXCN motif. Whether the partial TFD forms the disulfide bridges typically found within the complete TFD is unknown. The complexity of the carboxyl-terminus of the SPF-like contigs has not been determined and may contain the canonical disulfide bridges or may have low complexity similar to PPS. Based upon this domain structure and the cysteine pattern, a total of four complete and six partial SPF-like contigs were identified. Of the ten SPF-like contigs, nine closely followed the beta SPF structure. One SPF-like contig followed the alpha SPF structure, except its posterior cysteine motif contained only five cysteines instead of the canonical six cysteines (Fig 6).

The PMF-like homologs contained a signal peptide and a complete TFD and then terminated shortly following the CCXXXXCN motif at the carboxyl terminus of the TFD. The PMF-like homologs contained more amino acids between the cysteines and were longer than the canonical PMF. Additionally, the spacing between the cysteines varied from SPF. This domain structure and cysteine pattern was consistent with one complete PMF-like contig and two partial PMF-like contigs. One partial PMF-like contig extended 25 amino acids beyond the CCXXXXCN motif. AFP follows a domain structure similar to PMF [43] and has a moderate level of expression, suggesting that AFP may be involved in chemical communication along with PMF. We identified one complete AFP-like contig as well as four partial AFP-like contigs.

### Peptide pheromones are amongst the most abundantly expressed genes in the cloacal gland of an adult male axolotl

The program RSEM-eval revealed that peptide pheromones are among the most highly expressed genes in this individual wild-type male axolotl cloacal gland (Table 2) at the time of sacrifice. The top twenty most highly expressed genes included three forms of collagen (ranked 1, 3, and 4), two cell cycle checkpoint associated proteins (ranked 2 and 16), two ribosomal proteins (ranked 5 and 6), two forms of plasminogen (ranked 7 and 10), two forms of mucin (ranked 8 and 9), several uncharacterized proteins (ranked 11, 12, and 13), two cysteine-rich proteins (ranked 14 and 19), two forms of ferritin heavy chains (ranked 15 and 20), one complete SPF-like contig (ranked 17), and the complete PMF-like contig (ranked 18). Many of these most highly expressed genes make sense within the context of an axolotl cloaca. Collagen is among the most common proteins to be expressed in vertebrate cells [52], and the salamander cloaca secretes mucus as part of the spermatophore [53]. Generally, ribosomal proteins are expressed at very high levels relative to other proteins in most tissues [54]. Because the level of expression of this SPF-like contig (1477 TPMs) and this PMF-like contig (1336 TPMs) exceed most ribosomal proteins, these two pheromone proteins should be considered highly expressed. Additionally, we considered a contig with a TPM greater than 100 to be highly expressed; thus, these two contigs were considered very highly expressed. The other complete and partial SPF-like contigs ranged in expression from 2 TPMs to 235 TPMs with three partial SPF-like contigs exceeding 100 TPMs. The two partial PMF-like contigs were expressed at 2 TPMs. The complete AFP contig and two of the four partial AFP contigs were each expressed around 32 TPMs.

**Table 2:** BLAST matches of the most highly expressed contigs with a transcript per million (TPM) score greater than or equal to 1000 in the male axolotl cloacal gland transcriptome, listed from most highly expressed to least highly expressed.

Transcript ID	TPM	E-value	Species	Protein Description^§^
c3405_g1_i2	307839.2	3.9 x 10^−58^	*Rattus norvegicus*	Collagen alpha-1 chain-like
c3412_g1_i2	284003.8	4.1 x 10^−20^	*Xenopus tropicalis*	Chk1 checkpoint homolog
unc7258_g1_i1	81920.0	6.9 x 10^−6^	*Cavia porcellus*	Collagen alpha-1 chain-like
c3405_g1_i1	81032.7	5.4 x 10^−15^	*Rattus norvegicus*	Proline-rich protein HaeIII subfamily 1-like
unc2297_g1_i1	67435.5	1.6 x 10^−20^	*Manacus vitellinus*	40S ribosomal protein S29
c11288_g1_i1	29833.9	1.6 x 10^−12^	*Sorex araneus*	FAM228b-like protein
unc2267_g1_i1	9441.2	9.0 x 10^−8^	*Epinephelus bruneus*	Plasminogen
c3422_g1_i1	6545.2	1.2 x 10^−30^	*Xenopus tropicalis*	Integumentary mucin -like
c2358_g1_i1	4353.1	2.7 x 10^−180^	*Xenopus tropicalis*	Mucin-5B- partial
unc2554_g1_i1	3832.7	4.1 x 10^−20^	*Epinephelus bruneus*	Plasminogen
c4389_g1_i1	3677.0	1.1 x 10^−15^	*Sarcophilus harrisii*	Uncharacterized protein
unc757_g1_i1	3632.4	5.0 x 10^−6^	*Clostridium papyrosolvens*	Uncharacterized protein
unc2538_g1_i1	3110.5	2.8 x 10^−7^	*Strigamia maritima*	Uncharacterized protein
c1055_g1_i1	1774.6	6.6 x 10^−21^	*Elephantulus edwardii*	A-kinase anchor protein 13-like
c2453_g1_i1	1670.1	2.4 x 10^−114^	*Xenopus tropicalis*	Ferritin, heavy polypeptide 1
unc3333_g1_i1	1586.6	1.6 x 10^−20^	*Loa loa*	Senescence-associated protein
**c3350_g1_i1**	**1476.9**	**4.6 x 10**^**−11**^	***Xenopus tropicalis***	**SPF-like (Phospholipase A2 inhibitor subunit gamma B-like)**
**c2860_g1_i1**	**1336.2**	**3.8 x 10**^**−27**^	***Cynops pyrrhogaster***	**PMF-like (Sodefrin)**
c2847_g1_i1	1332.5	3.9 x 10^−52^	*Xenopus tropicalis*	Cysteine-rich secretory protein 3
c1304_g1_i1	1307.1	2.7 x 10^−113^	*Xenopus tropicalis*	Ferritin, heavy polypeptide 1

The putative homologs to SPF and PMF are bolded with their BLAST description in parentheses.

^§^ Raw descriptions from BLAST match

This study provides a first glimpse in determining relative expression levels, in a genome wide manner, by a relatively unbiased method [36]. Previous work used forward genetics approaches to identify biologically active molecules and gene sequences important in pheromone communication in salamanders. Recent studies have identified additional genes involved in the process, indicating a greater complexity than was previously appreciated. Reverse genetic approaches, like those used in this work, can help identify multiple genes with putative roles for pheromone communication. Future work may be able to leverage the combined value of each approach in providing a more complete picture of chemical communication in salamanders.

## Supporting Information

S1 Code Description of custom scripts. (DOCX)
