# Supplementary material for: Proteinaceous Pheromone Homologs Identified from the Cloacal Gland Transcriptome of a Male Axolotl, Ambystoma mexicanum
Source: PLoS One. 2016 Feb 17;11(2):e0146851. doi: 10.1371/journal.pone.0146851 (PMC4757389; doi:10.1371/journal.pone.0146851)
Supplement: S1 Code — (DOCX) [file pone.0146851.s001.docx]

This supplement provides an explanation of and rationale for the programs utilized in the assembly and annotation of the transcriptome to provide guidance for researchers with nominal prior experience in transcriptomics who are seeking to assemble a transcriptome of a non-model organism. Thus, the following code and program descriptions provide rudimentary guidance for novice transcriptome assemblers. The memory and time provide only a baseline for future work, but will vary from machine to machine and from program version to program version.

Code 1: Trimmomatic (v0.32) used four threads that required an additional core for a total request for ppn=5. Trimmomatic then took the original fastq file (WilliamsEisthen_ATCACG_L001_R1_001.fastq) and the file containing the Illumina adapters (illuminaClipping.fa:2:30:10) and removed the adapters from each sequence, creating a file of fastq sequences without the Illumina adapters (WEH.fastq.trimmed). The ‘WEH’ is for Williams , Eisthen, and Hall. This command took less than 10 min to execute using 2 GB of memory in the process.

Code 2: FaQCs (v1.3) is a perl script that analyzes the unpaired reads (-u WEH.fastq.trimmed) for reads (-avg_q 20) and bases (-q 20) with a target number of 20 as quality level for trimming. Eight threads (-threads) (ppn) were used and the prefix (-prefix) added to each of the output files was ‘WEH’. The trimmed reads were set to a minimum length of at least 35 (-min_L 35). Three files were created by this command: a pdf of graphics and reports (WEH_qc_report.pdf), a summary of the stats (WEH.stats.txt), and a FastQ file of the reads (WEH.unpaired.trimmed.fastq). FaQCs combines various features of comparable applications into a single, user-friendly process, where the original data and trimmed summaries are provided within a portable document format (pdf) containing an assortment of graphics and reports, enabling a simple assessment of data quality control. This command took less than 2 hr to execute using less than 1 GB of memory in the process.

Digital normalization removes highly redundant (high-coverage) reads from the billions of generated reads from the sequencer and normalizes average coverage. This reduces the total number of reads to be assembled and any sampling variation, as well as, the sequencing errors contained within removed reads [1]. A k-mer is a nucleotide polymer of ‘k’ length.

Code 3: Digital normalization used screed (v0.7) [1] and khmer (v1.3) [2] to take the filtered reads from outputted by FaQCs from Code 2 and limits the number of redundant reads to 20 by discarding sequences based on whether or not their median k-mer abundance lies above the specified cutoff (-C 20). A k-mer is a nucleotide sequence of ‘k’ length. The k-mer length specified here is twenty (-k 20). Four k-mer counting tables (-N 4) were used, each with a hash table size of 2x10^9^ (-x 2e9). The WEH.fq.keep file was created containing the reads from WEH.unpaired.trimmed.fastq that were not discarded due to overly high coverage. This command took less than 2 hr to execute using approximately 8 GB of memory in the process.

Trinity can be broken down into five individual stages. This compartmentalization enabled faster assembly due to shorter wait times on the high performance computing cluster without impacting the quality of the transcriptome assembly (Code 4). Trinity has three main components, “Inchworm”, “Chrysalis”, and “Butterfly”. The Inchworm component assembles the filtered reads by extending the reads through k-mer space to create a set of contigs, where each read is used no more than once. Next, the Chrysalis component groups contigs that share at least (k-1)-mer bases, and if reads span the junction between contigs, it then builds individual de Bruijn graphs for each group. Finally, the Butterfly component takes the de Bruijn graphs from Chrysalis and removes unlikely paths resulting in more compact graphs. It then reconciles the de Bruijn graph with reads and outputs one linear sequence for each alternatively spliced transcript represented in the de Bruijn graph [3]. Each stage of Trinity required the initial filtered single-end reads and output directory indicating where to save files created while Trinity ran. The minimum isomer ratio parameter setting means that 10% of one inchworm contig must align to another inchworm contig in order for the two contigs to be put together as one larger contig. The low glue factor parameter setting means fewer reads are required to support the joining of inchworm contigs, resulting in a greater likelihood that contigs are discovered, but also results in a greater number of false contigs [4]. The minimum contig length parameter sets the minimum length of contigs in the final Trinity.fasta file to 200 bp. The final parameter specifies how much memory the subprogram Jellyfish should use. In order to compartmentalize each stage, a separate parameter was added to each coding sequence. These parameters would indicate where Trinity should end. With each subsequent stage, Trinity would check to see what files are present and automatically pick up the program where it left off from previous stage. The first stage generates the kmer-catalog using the program ‘Jellyfish’ and stopped prior to Inchworm beginning (Code 4a). Upon completion of the kmer-catalog, Inchworm was executed and stopped prior to running Chrysalis (Code 4b). The third step began to run the Chrysalis component by clustering the Inchworm contigs and mapping reads, but stopping before graph quantification (Code 4c). The fourth step finished Chrysalis by creating the de Bruijn graphs and stopping before Butterfly (Code 4d). The fifth and final step excludes any --no_ options and runs Butterfly to generate the final Trinity.fasta file (Code 4e). At this point, a Fasta file of a set of putative contigs was created and renamed ’WEH.fasta’.

Code 4: The ability of trinity to be compartmentalized enables faster shorter wait times on high-performance computing clusters. For each step, the glue factor was set to 0.01 (--glue_factor 0.01), the minimum isomer ratio was set to 0.1 (--min_iso_ratio 0.1), the minimum contig length was set to 200 base pairs (--min_contig_length 200) and the jellyfish memory was set to 48 gigabytes (--JM 48G). The first stage required the initial filtered single-end reads (--single /path/WEH.fq.keep) and output directory (--output /path/trin_WEH) to generate the kmer-catalog using the program Jellyfish and stopped prior to Inchworm beginning (--no_run_inchworm) (a). This first step took less than 10 min to execute using less than 40 GB of memory. Upon completion of the kmer-catalog, Inchworm was executed and stopped prior to running Chrysalis (--no_run_chrysalis) (b). This second step took approximately 1 hr to execute, using 17 GB of memory. The third step began to run the Chrysalis component by clustering the Inchworm contigs and mapping reads, but stopping before graph quantification (--no_run_quantifygraph) (c), taking approximately 45 min in the process and using less than 4 GB of memory. The fourth step finished Chrysalis by creating the de Bruijn graphs and stopping before Butterfly (--no_run_butterfly) (d). This step took less than 10 min and less than 1 GB of memory. The fifth step excludes any --no_ options and runs Butterfly to generate the final Trinity.fasta file (e). This step took less than 2 hr to execute using less than 5 GB of memory in the process.

TransDecoder is packaged with the Trinity program and has several components to maximize the likelihood of identifying putative ORFs (Code 5).

Code 5: In order to identify the maximum number of putative protein coding regions, (a) TransDecoder first searches for the longest ORFs in the list of contigs (-t WEH.fasta) that are at least 125 amino acids in length (-m 125) and outputs them into longest_orfs.pep. (b) BLASTp [5] broke this list of putative proteins (-query longest_orfs.pep) into eight individual threads for eight computer cores (-num_threads 8) to search for alignments within the UniProKB Swiss-Prot database (-db uniprot_sprot.fasta) and returns the top one target sequence (-max_target_seqs 1) with an e-value greater than 10^-5^ (-evalue 1e-5) in a tabular output format (-outfmt 6 -out blastp_WEH_uniprot.outfmt6). (c) Next, the list of putative proteins (longest_orfs.pep) is searched for protein families found within the pfam database (Pfam-AB.hmm) to create a tabular output of putative pfam domains. (d) Finally, TransDecoder runs predictions about which contigs (-t WEH.fasta) are proteins using the information from the longest ORFs, BLASTp results (--retain_blastp_hits blastp_WEH_uniprot.outfmt6) and identified pfam domains (--retain_pfam_hits pfam_WEH.domtblout). This process required about 6 hr to execute and used less than 2 GB of memory in the process.

The Basic Local Alignment Search Tool (BLAST) allows for the searching of databases for sequences to which the putative contigs align [5]. BLASTx takes nucleotide sequences and translates them into all six frames and then takes those protein sequences to search for alignments against a protein database. BLASTp takes amino acid sequences and searches for alignments against a protein database.

Code 6: BLASTx (a-c) or BLASTp (d-f) divided this list of contigs (-query WEH.fasta) into eight individual threads for eight computer cores (-num_threads 8) to search for alignments within the *X. tropicalis* protein database (a), UniRef90 protein database (b), UniProKB Swiss-Protein database (c) and returns the top one target sequence (-max_target_seqs 1) with at least an e-value greater than 10^-5^ (-evalue 1e-5) in a tabular output format (-outfmt 6). The time and memory requirements for BLAST to function depend upon the size of the query and the size of the database. When querying the *X. tropicalis* protein database, the time and memory requirements are much lower because the database contains only proteins from *X. tropicalis*, finishing within 1 hr and utilizing less than 2 GB of memory, while the UniRef90 database may require up to half a day to complete and 15 GB of memory.

Code 7: Bowtie2 takes reads and aligns the reads against a reference genome. In this case, the filtered fasta transcriptome (WEH_filtered.fasta) sequences are used as the reference genome. Bowtie2-build creates a Bowtie index from the transcriptome sequences, outputting six files with an assigned basename (WEH_filtered), which are required to align the filtered reads to the reference transcriptome (a). The Bowtie2 program utilizes seven cores (-p 7, but note that because of the execution method the program uses, you will need one additional core available. For the HPCC at MSU, the PPN was set to 8 even though the program requests 7) to align the single-ended filtered reads (-U WEH. unpaired.trimmed.fastq) from FaQCs.pl (Code 2) to the index files of the reference genome (-x WEH_filtered) (b). Bowtie2 aligns the reads end to end, preventing any soft-clipping or trimming of the reads (--end-to-end). Bowtie2 can also create a sequence alignment/map file (-S WEH_filtered_align.sam) and parses the unaligned reads (--un) from the aligned reads (--al). This process took less than 1 hr to execute and less than 1 GB of memory.

Code 8: RSEM-eval first calculates the average transcript length (676.2) and the standard deviation (1246.3) of the multi-fasta complete filtered transcriptome (WEH_filtered_total.fasta) and outputs the calculations to a parameter file (para_filt_tot_WEH.eval) (a). Then RSEM-eval utilizes eight threads (-p 8) and Bowtie2 (--bowtie2) to estimate the level of expression for each transcript by mapping the filtered reads (WEH.unpaired.trimmed.fastq) to the complete filtered transcriptome (WEH_filtered_total.fasta) when the read length is specified (50). Each outputted file and directory has the assigned prefix (filt_tot_WEH_Score). RSEM-eval can also generate a BAM file (--output-bam) and calculate the confidence intervals (--calc-ci) for the Transcripts Per Million (TPM). This process required about 5 hr and 16 GB of memory to execute.

Code 9: Each nucleotide sequence was run through interproscan (a) and BLASTx (b-f). Each peptide sequence was run through BLASTp (g-k). The term ‘xx’ refers to the numeric value assigned to the fasta sequence file by ‘split_fasta.pl’. The amount of time required depended on the length of the sequence. For most sequences, the time was less than 1 hr, however there were a number of sequences that required 1-6 hr. All sequences needed at least 15 GB of memory due to the size of the protein databases utilized.

1. Brown CT, Howe A, Zhang Q, Pyrkosz AB, Brom TH. A reference-free algorithm for computational normalization of shotgun sequencing data. arXiv preprint arXiv:12034802. 2012.

2. Crusoe M, Edvenson G, Fish J, Howe A, McDonald E, Nahum J, et al. The khmer software package: enabling efficient sequence analysis. URL <http://dx> doi org/106084/m9 figshare. 2014;979190.

3. Grabherr MG, Haas BJ, Yassour M, Levin JZ, Thompson DA, Amit I, et al. Full-length transcriptome assembly from RNA-Seq data without a reference genome. Nat Biotechnol. 2011;29(7):644-52. doi: 10.1038/nbt.1883. PubMed PMID: 21572440; PubMed Central PMCID: PMC3571712.

4. Li B, Fillmore N, Bai Y, Collins M, Thomson JA, Stewart R, et al. Evaluation of de novo transcriptome assemblies from RNA-Seq data. Genome Biol. 2014;15(12):553. doi: 10.1186/s13059-014-0553-5. PubMed PMID: 25608678; PubMed Central PMCID: PMC4298084.

5. Altschul SF, Gish W, Miller W, Myers EW, Lipman DJ. Basic local alignment search tool. Journal of molecular biology. 1990;215(3):403-10.
